# Acoustic Noise Levels in High‐field Magnetic Resonance Imaging Scanners

**DOI:** 10.1002/oto2.79

**Published:** 2023-09-18

**Authors:** Armaan F. Akbar, Zahra N. Sayyid, Dale C. Roberts, Jun Hua, Adrian Paez, Di Cao, Amanda M. Lauer, Bryan K. Ward

**Affiliations:** ^1^ Department of Otolaryngology–Head and Neck Surgery Johns Hopkins University School of Medicine Baltimore Maryland USA; ^2^ Department of Neurology Johns Hopkins University School of Medicine Baltimore Maryland USA; ^3^ F.M. Kirby Research Center for Functional Brain Imaging Kennedy Krieger Institute Baltimore Maryland USA; ^4^ Department of Radiology Johns Hopkins University School of Medicine Baltimore Maryland USA; ^5^ Department of Neuroscience Johns Hopkins University School of Medicine Baltimore Maryland USA

**Keywords:** 3‐Tesla MRI, 7‐Tesla MRI, acoustic noise, brain imaging, inner ear imaging

## Abstract

7‐Tesla (T) magnetic resonance imaging may allow for higher resolution images but may produce greater acoustic noise than 1.5‐ and 3‐T scanners. We sought to characterize the intensity of acoustic noise from 7‐ versus 3‐T scanners. A‐weighted sound pressure levels from 5 types of pulse sequences used for brain and inner ear imaging in 3‐ and 7‐T scanners were measured. Time‐averaged sound level and maximum sound levels generated for each sequence were compared. Time‐averaged sound levels exceeded 95 dB and reached maximums above 105 dB on the majority of 3‐ and 7‐T scans. The mean time‐averaged sound level and maximum sound level across pulse sequences were greater in 7‐ than 3‐T (105.6 vs 91.4, *P* = .01; 114.0 vs. 96.5 dB, *P* < .01). 7‐ and 3‐T magnetic resonance imaging scanners produce high levels of acoustic noise that exceed acceptable safety limits, emphasizing the need for active and passive noise protection.

Acoustic noise from magnetic resonance imaging (MRI) can cause patient anxiety and discomfort, hearing damage, and impaired communication between patients and health‐care workers.^1^, ^2^, ^3^, ^4^ This noise is generated by interactions between the static magnetic field and alternating current in gradient coils, resulting in Lorentz forces that vibrate the coils, creating mechanical noise. Lorentz forces increase with magnetic field strength and electrical current in gradient coils. MRI using 1.5‐Tesla (T) and 3 T magnetic fields as well as increasingly powerful gradient electronics exposes patients to sound pressure levels (SPL) as high as 130 decibels (dB SPL),^5^ nearing the maximum allowable SPL of 140 dB as set by the United States Food and Drug Administration (FDA).^6^

7 T MRI with optimized pulse sequences may improve image resolution of the brain and inner ear, but may produce even greater acoustic noise than 1.5 and 3 T scanners.^7^ Louder noises can result in temporary or permanent hearing loss; therefore, the sound intensity of noise generated in 7 T MRI needs to be better understood. In this study, we sought to characterize the intensity of acoustic noise generated in 7 and 3 T MRI scanners.

## Methods

SPLs and spectra during MRI scans in 3 and 7 T Phillips scanners (Phillips Healthcare) were measured using a Larson Davis LxT digital sound level meter (SLM) (Larson Davis). SPLs from 5 types of pulse sequences commonly used for brain and inner ear imaging were recorded (Table 1). A 12.7‐mm microphone (PCB Piezoelectronics model 375A02) was positioned at the scanner's isocenter, oriented along the *z*‐axis, and attached to the SLM. Overall SPLs and 1/3 octave band levels (center frequencies of 20 Hz to 20 kHz) were measured using the A‐weighting function, which approximates the human ear response to noise. SPLs were recorded every 0.125 seconds as time‐averaged sound level (LAeq) and maximum sound level (LAmax); LAeq is the energy equivalent level calculated over the measurement time period for the summation of all frequency bands; LAmax is the highest level detected during the measurement period. The SLM calculates LAeq from A‐weighted SPLs according to the following equation: ${\mathrm{LAeq}}_{T}=201g\{{\left[\left(1/T\right)\int_{t-T}^{\tau }P_{A}^{2}\left(\xi \right)d\xi \right]}^{1/2}/P_{0}\}\mathrm{dB}$.^8^ The study protocol was approved by the Johns Hopkins University School of Medicine Institutional Review Board (NA00041628).

**Table 1:** Pulse Sequence Descriptions and Parameters for 3 T and 7 T Pulse Sequences Used

	Sequence	Function	TR (ms)	TE (ms)	FOV (mm)	Scan Resolution *x*/*y*/*z* (mm)	# Slices/3D	# Echoes	Coil
3 T	Survey	Survey to view brain location in the scanner	9.8	4.6	250 × 250 × 50	0.98/1.95/1	3 orientations, 3 slices per stack	1	32‐channel head coil
	T1w Fast Field Echo (FFE)	Imaging	15	3.9	210 × 183 × 150	0.8/0.8/0.8	3D	1	32‐channel head coil
	Multi‐Echo Turbo Spin Echo (TSE)	Imaging	6000	676	212 × 170 × 20	1/1/1	2D Multi slice, 20 slices	8	32‐channel head coil
	T2w TSE	Imaging	3000	85	230 × 181 × 189	0.8/0.8/0.8	2D Multi slice, 237 slices	1	32‐channel head coil
	3D Fluid‐attenuated Inversion Recovery (FLAIR)	Imaging	6000	2000	210 × 183 × 150	0.8/0.8/0.8	3D	1	32‐channel head coil
7 T	Multi‐Slice FFE, 3 plane	Survey to view brain location in the scanner	11	4.9	250 × 250 × 50	0.98/1.95/1	3 orientations, 3 slices per stack	1	8‐channel multi‐transmit coil
	Magnetization prepared rapid gradient‐echo (MPRAGE)	Imaging	5	1.8	220 × 220× 180	1/1/1	3D	1	8‐channel multitransmit coil
	T1w Spin Echo	Imaging	600	10	230 × 183 × 4	0.9/1.12/4	1 slice	1	8‐channel multitransmit coil
	3D T2w Spin Echo	High resolution imaging	3862	193	180 × 150 × 8	0.3/0.3/0.3	3D	1	8‐channel multitransmit coil
	3D FLAIR	Imaging	8000	420	205 × 205 × 9	0.6/0.6/0.8	3D	1	8‐channel multitranslmit coil

## Results

LAeq exceeded 95 dB and reached maximums above 105 dB for the majority of scans at 3 T and 7 T (Table 2). LAeq were greater in the 7 T than the 3 T scanner for each pulse sequence type assessed except T1‐weighted spin echo. The loudest 3 T sequence was T1‐weighted spin echo, reaching a maximum of 104.8 dB; the loudest 7 T sequence was T2‐weighted spin echo, reaching 121.6 dB. The mean LAeq and LAmax across pulse sequences was higher in 7 T than 3 T for LAeq (105.6 vs. 91.4, *P* = .01) and for LAmax (114.0 vs 96.5 dB, *P* < .01).

**Table 2:** LAeq and Peak Measurements in dB

	LAeq			LAmax		
	3 T	7 T	*P* value	3 T	7 T	*P* value
Survey	89.4	101.8		96.0	106.6	
T1 Gradient Echo	88.9	114.4		90.9	120.4	
T1 Spin Echo	100.9	97.5		104.8	105.5	
T2 Spin Echo	96.6	110.1		96.8	121.6	
FLAIR	81.3	104.1		94.0	115.7	
Mean (SD)	91.4 (7.6)	105.6 (6.7)	.014*	96.5 (5.2)	114.0 (7.6)	.003*

* *P* < .05.

**Figure 1:**
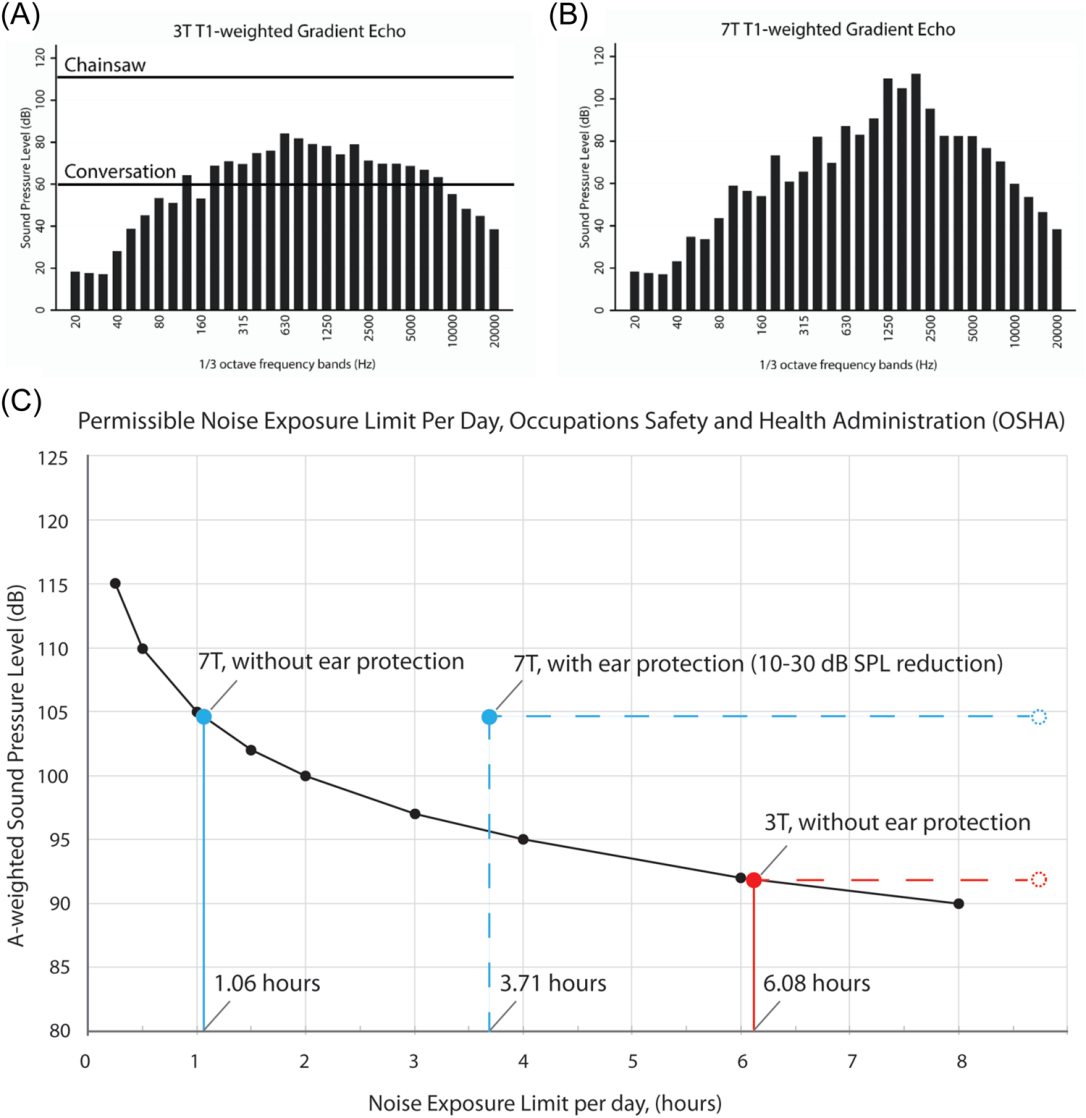
A‐weighted MRI acoustic noise for a sample T1‐weighted sequence (A, B) and study average A‐weighted SPL at 3 T (red) and 7 T (blue) with (dotted line) and without (solid line) hearing protection, versus permissible noise exposure limits by OSHA (C). MRI, magnetic resonance imaging; OSHA, Occupational Safety and Health Administration

A‐weighted MRI acoustic noise for all pulse sequences is shown in Figure 1 and Supplemental Figure S1, available online. With hearing protection, sound levels for the duration of clinical scans were within permissible noise exposure limits set by the Occupational Safety and Health Administration.

## Discussion

The United States FDA allows a maximum SPL of 140 dB and LAeq of 99 dB—with hearing protection—for safe MRI use.^6^ This study found that 7 T and 3 T MRI scanners produce levels of acoustic noise exceeding the recommended maximum during common scanning protocols for imaging the brain and inner ear. Prior reports hypothesized that 7 T and 3 T may produce similar noise^9^, ^10^; however, we found that 7 T scans were louder on average, consistent with prior studies in 0.2 to 3 T scanners.^11^ Greater magnetic field strength increases the force that vibrates MRI gradient coils in accordance with Lorentz' law, generating more intense noise.^12^ Noise from 7 T and 3 T scanners poses health risks to patients and providers in clinical and research settings.

Well‐fitting ear plugs provide an easy, low‐cost form of passive protection that can attenuate noise by 10 to 30 dB.^13^ While this may decrease the observed noise to acceptable levels, cochlear function can be affected during MRI even with passive protection, and ear plugs alone may be insufficient.^14^ Since the acoustic noise produced by MRI is mainly due to the mechanical vibrations from the switching of electric currents within gradient coils,^15^ sequence‐based approaches can alter gradient activity and actively reduce noise.^16^ Quiet sequences for 3 T scanners resulting in reductions of 20 to 40 dB have been described,^17^, ^18^, ^19^, ^20^ and a recently developed quiet 7 T FLAIR sequence reported a 14 dB reduction.^21^

This study has limitations. SPLs after attenuation from passive or active noise protection strategies were not measured, so they may not reflect the noise that patients would experience in clinical practice. Noise may reach the human ear differently than that measured by our SLM, and system loading can cause noise variations up to 10 dB.^7^ Additionally, SPLs produced by two Phillips scanners were measured; MRI hardware and pulse sequence variation can contribute to differences in noise production. Nevertheless, this study suggests that 7 T and 3 T MRI produce high levels of acoustic noise and highlights the importance of hearing protection during MRI.

## Conclusion

This study found that 7 T and 3 T MRI produce high levels of acoustic noise that exceed acceptable safety limits, emphasizing the need for active and passive noise protection.

## Author Contributions

**Armaan F. Akbar**, BS, collected data, analyzed data, presented the data, and wrote the initial manuscript draft; **Zahra N. Sayyid**, MD, PhD, analyzed data and wrote the initial manuscript draft; **Dale C. Roberts**, MS, designed the study and collected data; **Jun Hua**, PhD, designed the study; **Adrian Paez**, BS, collected data; **Di Cao**, PhD, collected data; **Amanda M. Lauer**, PhD, designed the study, reviewed the data, and edited the manuscript; **Bryan K. Ward**, MD, designed the study, collected data, and edited the manuscript. All authors approved the final version of the manuscript.

## Disclosures

### Competing interests

The authors declare that there is no conflict of interest.

### Funding sources

NIH K23DC018302, the David M. Rubenstein Fund for Hearing Research, and the Robert and Kate Niehaus Foundation.

## Supporting information


**Supplemental Figure 1**. A‐weighted MRI acoustic noise. Spectra were generated by separate Survey (A, B), T1‐weighted Spin Echo (C, D), and FLAIR (E, F) sequences in 3T (A, C, E) and 7T (B, D, F) scanners.
