# Supplementary figures and images for: Acoustic Noise Levels in High‐field Magnetic Resonance Imaging Scanners
Source: OTO Open. 2023 Sep 18;7(3):e79. doi: 10.1002/oto2.79 (PMC10506133; doi:10.1002/oto2.79)

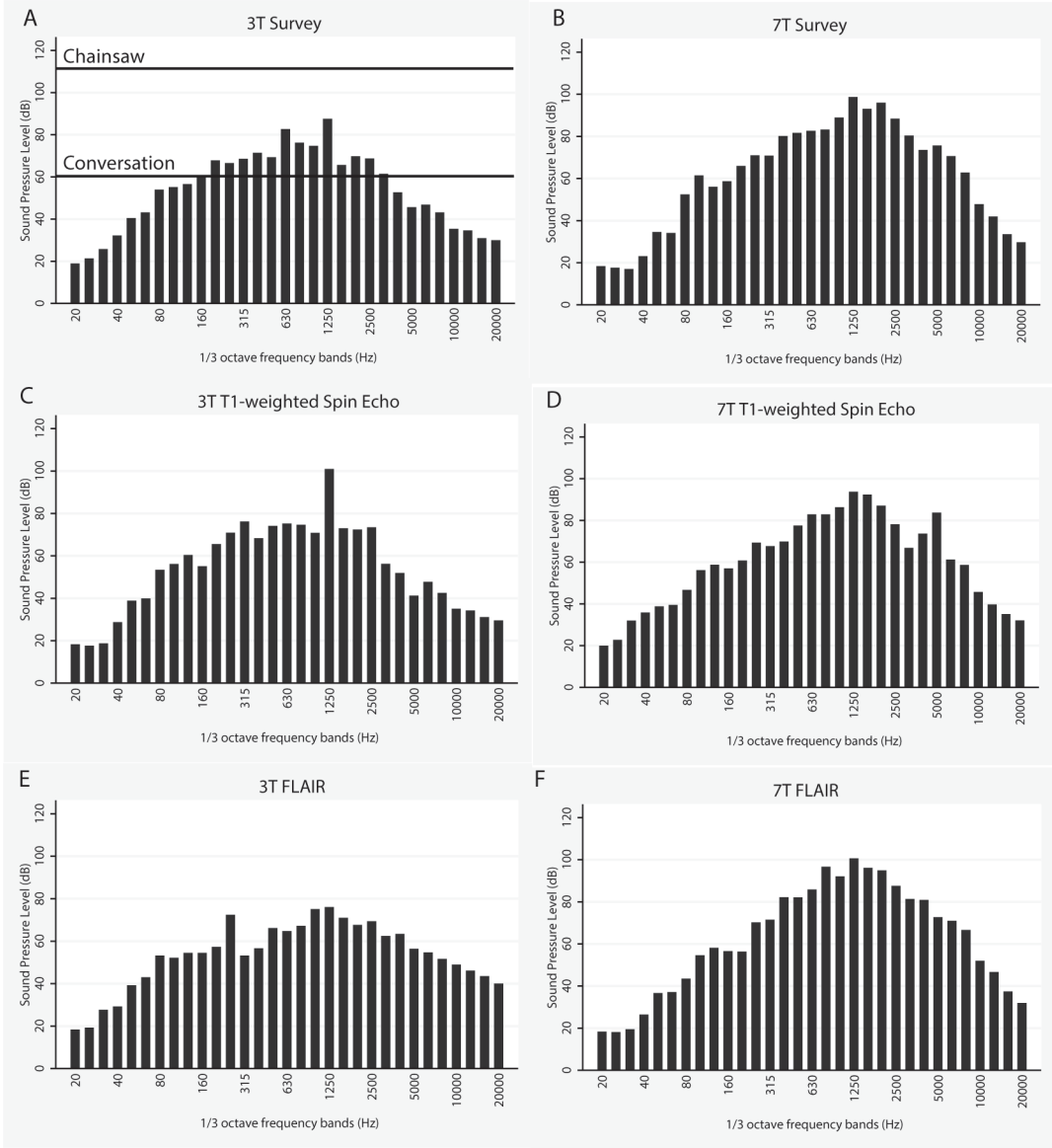

Supplement: Supplementary file 1 — Supplemental Figure 1. A‐weighted MRI acoustic noise. Spectra were generated by separate Survey (A, B), T1‐weighted Spin Echo (C, D), and FLAIR (E, F) sequences in 3T (A, C, E) and 7T (B, D, F) scanners. [file OTO2-7-e79-s001.pdf]
